# Evaluation of Intraprocedural Strategies to Assess Acute Pulmonary Vein Isolation After First-Pass Wide Antral Circumferential Ablation

**DOI:** 10.3390/jcm15197654

**Published:** 2026-10-02

**Authors:** Lyuboslav Katov, Julian Ballweg, Yannick Teumer, Jonas Rostan, Federica Diofano, Carlo Bothner, Wolfgang Rottbauer, Karolina Weinmann-Emhardt

**Affiliations:** Department of Cardiology, Ulm University Heart Center, Albert-Einstein-Allee 23, 89081 Ulm, Germany; lyuboslav.katov@uniklinik-ulm.de (L.K.); julian.ballweg@uni-ulm.de (J.B.); yannick.teumer@uniklinik-ulm.de (Y.T.); jonas.rostan@uniklinik-ulm.de (J.R.); federica.diofano@uniklinik-ulm.de (F.D.); carlo.bothner@uniklinik-ulm.de (C.B.); wolfgang.rottbauer@uniklinik-ulm.de (W.R.)

**Keywords:** radiofrequency ablation, pulmonary vein isolation, wide antral circumferential ablation, 3D electroanatomical mapping

## Abstract

**Background**: Atrial fibrillation (AF) is the most common sustained cardiac arrhythmia. While pulmonary vein isolation (PVI) remains the cornerstone of AF ablation, the optimal method for confirming complete acute isolation remains uncertain. This study evaluated the diagnostic value of pulmonary vein (PV) pacing compared with repeat three-dimensional (3D) electroanatomical mapping following wide antral circumferential ablation (WACA). **Methods**: In this prospective observational study, 58 patients with paroxysmal or persistent AF undergoing bilateral WACA at the University Heart Center Ulm between November 2023 and April 2025 were included. Following successful PVI, pacing was performed from each PV and the corresponding carina region using a maximal stimulation output of 20 mA. Subsequently, comprehensive 3D re-mapping was performed to reassess electrical conduction. Results obtained from pacing maneuvers were compared with repeat mapping findings. **Results:** The study population had a median age of 73 (62.7–78.2) years and a median body mass index of 26.8 (24.0–29.9) kg/m^2^. Median left atrial diameter was 4.8 (4.3–5.5) cm, and moderate to advanced atrial fibrosis was present in approximately one-third of patients. For the left-sided PVs, pacing assessment alone was associated with a significantly higher proportion of false-negative results compared with 3D re-mapping (left upper PV: *p* = 0.039; left inferior PV: *p* = 0.012). In contrast, no significant differences were observed for the right PVs. Evaluation of the carina regions showed a similar pattern, with a significant discrepancy on the left side (*p* = 0.002) but not on the right side (*p* = 0.453). **Conclusions**: PV pacing alone may not reliably identify residual conduction within the left PVs and left carina after WACA. Additional 3D re-mapping appears valuable in these regions to detect persistent conduction gaps that could otherwise remain unnoticed. For right-sided PVs, pacing-based assessment may provide sufficient confirmation of acute isolation in many cases. These findings support a complementary use of both techniques to improve procedural verification of PVI. Larger prospective studies are warranted to confirm the observed results and determine their clinical implications.

## 1. Introduction

Atrial fibrillation (AF) is the most common sustained cardiac arrhythmia, affecting more than 50 million people worldwide. Its prevalence continues to rise as a result of population aging, improved detection methods, and the increasing burden of cardiovascular risk factors [1,2,3]. AF is associated with a significantly heightened risk of stroke and heart failure, impaired quality of life, and higher all-cause mortality [4].

Following the groundbreaking study by Haïssaguerre et al., pulmonary vein isolation (PVI) has become the cornerstone ablation strategy for AF [5]. This pivotal insight accelerated progress in AF ablation technologies, ultimately contributing to the widespread adoption of three-dimensional (3D) electroanatomical mapping (EAM) systems and radiofrequency ablation (RFA) in modern electrophysiological practice [6,7,8]. However, robust evidence identifying the most reliable approach to evaluate acute PVI during the RFA procedure is currently unavailable. Conventional assessment commonly relies on entrance and exit block verification at the pulmonary vein (PV) ostia following ablation. However, this approach may fail to identify residual slow conduction or areas susceptible to reconnection, potentially compromising long-term procedural success [9,10].

The present study aims to compare pacing maneuvers performed exclusively at the PV ostia with a combined remapping and pacing approach to identify residual or potentially vulnerable conduction after first-pass wide antral circumferential ablation (WACA). By systematically evaluating these intraprocedural strategies, we seek to determine the most reliable method for assessing acute PVI and optimizing procedural outcomes.

## 2. Materials and Methods

### 2.1. Trial Population

Between November 2023 and April 2025, 58 consecutive patients were prospectively enrolled at the Ulm University Heart Center. Patients with AF scheduled for wide circumferential PVI guided by 3D EAM and RFA were considered eligible for inclusion. Exclusion criteria included patients younger than 18 years as well as those undergoing ablation of other left atrial tachycardias, such as focal atrial tachycardia or atypical atrial flutter. Prior to the intervention, all patients provided written informed consent. The study protocol was reviewed and approved by the Ethics Committee of the University of Ulm and adhered to the ethical standards of the Declaration of Helsinki. Data acquisition was carried out prospectively as part of the ATRIUM registry (German Clinical Trials Register ID: DRKS00013013).

### 2.2. Periprocedural Management and Verification of PVI After Bilateral WACA

Deep sedation was achieved using a standardized protocol consisting of an initial propofol bolus (0.5 mg/kg body weight, reduced by 10% in patients with impaired left ventricular ejection fraction), followed by continuous, individually adjusted propofol infusion (2–6 mg/kg/h). Analgesia was provided by concomitant remifentanil administration (0.05–0.2 µg/kg/min). After induction of sedation, transesophageal echocardiography was performed to exclude intracardiac thrombus and to support intraprocedural imaging guidance. For esophageal temperature monitoring during ablation of the posterior left atrial wall, a multi-electrode temperature probe was placed transnasally. Oral anticoagulation therapy was not discontinued prior to ablation, and intraprocedural unfractionated heparin (UFH) was administered to achieve an activated clotting time (ACT) between 300 and 400 s. A comprehensive description of our procedural workflow has been published previously [11].

**Figure 1:**
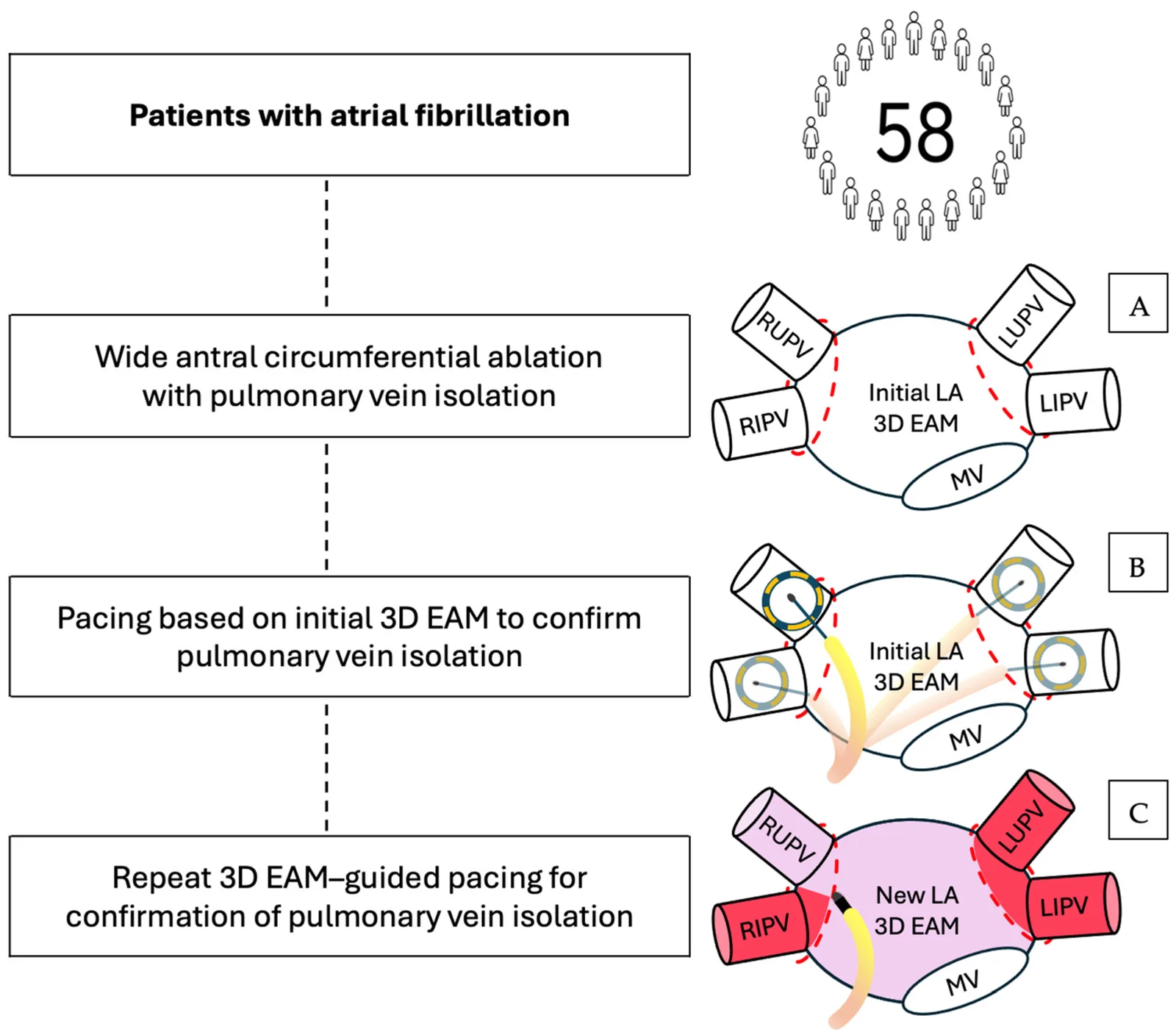
Schematic overview of the study design and procedural workflow, including wide antral circumferential ablation, pacing, and repeat three-dimensional electroanatomical mapping with pacing to confirm complete pulmonary vein isolation. All figures depict the left atrium in an anterior view. (**A**) illustrates the initial wide antral circumferential ablation, indicated by a red dashed line encircling the pulmonary veins. (**B**) demonstrates the sequential pacing maneuvers performed at all pulmonary vein ostia distal to the ablation line. (**C**) shows repeat electroanatomical maps revealing, for example, reconnection of the right upper pulmonary vein and subsequent re-ablation to achieve complete isolation. In this panel, isolated pulmonary veins are schematically highlighted in red, whereas residual electrical signals are depicted in purple. In (**B**,**C**), the transseptal sheath is also visible and marked in yellow. Three-dimensional EAM, three-dimensional electroanatomical mapping; LA, left atrium; LIPV, left inferior pulmonary vein; LUPV, left upper pulmonary vein; MV, mitral valve; RIPV, right inferior pulmonary vein; RUPV, right upper pulmonary vein.

For 3D EAM and RFA, three femoral venous access sites were obtained in the right groin under ultrasound guidance. A steerable decapolar diagnostic catheter (Inquiry™ Steerable Diagnostic Catheter, Abbott, North Chicago, IL, USA) was introduced into the coronary sinus via a 7-F femoral sheath. Transseptal puncture was performed using a non-steerable sheath (CardiaGuide™, Johnson & Johnson, New Brunswick, NJ, USA) in conjunction with a dedicated transseptal puncture needle (HeartSpan™ Transseptal Needle™, Johnson & Johnson, New Brunswick, NJ, USA). Upon left atrial access, the non-steerable sheath was exchanged for a steerable sheath (Vizigo™, Johnson & Johnson, New Brunswick, NJ, USA, or Agilis™, Abbott, Chicago, IL, USA) and positioned in the left upper PV (LUPV) under fluoroscopic and transesophageal echocardiographic guidance. Detailed 3D EAM of the left atrium was then carried out using either the Carto™ (Johnson & Johnson, New Brunswick, NJ, USA) or Rhythmia™ (Boston Scientific, Marlborough, MA, USA) mapping system, employing the OctaRay™ or PentaRay™ (Johnson & Johnson, New Brunswick, NJ, USA) and the Orion™ (Boston Scientific, Marlborough, MA, USA) mapping catheters, respectively. All left atrial maps were acquired during sinus rhythm using coronary sinus electrograms as the temporal reference. Bipolar voltage maps were displayed using lower and upper voltage thresholds of 0.1 and 0.5 mV, respectively, and each map comprised at least 1000 EAM points. Atrial fibrosis was categorized into four grades: grade 0, no detectable fibrosis; grade I, mild fibrosis; grade II, moderate fibrosis; and grade III, advanced fibrosis. PVI was achieved by bilateral WACA guided by the acquired EAM. Ablation was performed using either the SmartTouch SF™ or QDot™ catheter (Johnson & Johnson, New Brunswick, NJ, USA) or the StablePoint™ catheter (Boston Scientific, Marlborough, MA, USA). For conventional RFA, a power setting of 50 W was used, with applications of 15 s at the anterior wall and 10 s at the posterior wall. In procedures performed with the QDot™ catheter, 90 W/4 s applications were used for the initial WACA at the left posterior wall and around the right PVs. Lesions were applied contiguously along the WACA line with an interlesion distance of 3–5 mm under 3D EAM guidance. If additional ablation was required after remapping, RF energy was delivered at 50 W irrespective of anatomical location. No predefined waiting period was applied after completion of the initial WACA. After completion of the initial WACA, pacing maneuvers were performed distal to the ablation line and in close proximity to it. Pacing was applied from all PVs as well as from the carina regions to evaluate for residual conduction and to confirm effective electrical isolation. It was systematically performed at an output of 20 mA with a cycle length of 500 ms at four predefined sites around each PV ostium, including the superior, inferior, anterior, and posterior aspects, as well as at the anterior and posterior aspects of each ipsilateral carina. Local myocardial capture alone was not considered indicative of residual conduction if exit block was preserved. Complete bidirectional isolation required the presence of both entrance and exit block. For the purposes of this study, residual conduction was documented only when neither entrance nor exit block could be demonstrated, indicating persistent conduction in both directions. All findings obtained during the initial pacing-based assessment were documented according to the study protocol before subsequent EAM remapping was initiated. Subsequently, a detailed remapping of the left atrium was performed, with particular focus on the PVs and the bilateral carina regions. Electrograms suggestive of residual conduction were reviewed to exclude far-field left atrial appendage or ventricular signals based on electrogram morphology and timing relative to the reference electrograms. Suspicious or ambiguous mapping findings were further evaluated by targeted pacing, initially using the mapping catheter and subsequently the ablation catheter. This approach was defined as mapping-based assessment. In cases of incomplete PV or carina isolation, the affected sites were documented according to our study protocol, and additional ablation was performed, followed by repeat remapping to verify completeness (Figure 1).

### 2.3. Statistical Analysis

Statistical analyses were performed using SPSS^®^ Statistics software (version 29.0.1.0, IBM, Armonk, NY, USA). Continuous variables were summarized as medians with interquartile ranges (IQR), whereas categorical variables were presented as absolute numbers and percentages. Paired comparisons between pacing-based and mapping-based assessment of residual conduction were performed separately for each anatomical region using the exact McNemar test, thereby accounting for the paired nature of the two assessment strategies within each region. The McNemar test was interpreted as a measure of asymmetry in paired classifications rather than diagnostic superiority, as neither assessment strategy constituted an independent reference standard. Agreement between both assessment strategies was evaluated using overall percentage agreement and Cohen’s κ coefficient with corresponding 95% confidence intervals. Cohen’s κ was interpreted as a measure of agreement between the two assessment strategies and not as a measure of diagnostic accuracy. To account for multiple comparisons across the six anatomical regions, McNemar *p* values were additionally adjusted using the Holm method. Given the relatively small number of discordant observations in some regions, regional agreement estimates and their confidence intervals were interpreted descriptively. All statistical tests were two-sided, and a *p* value < 0.05 was considered statistically significant.

## 3. Results

### 3.1. Baseline Characteristics

A total of 58 patients were enrolled in this study. The median age was 73 (62.7–78.2) years, and the majority of patients were male (67.2%). The cohort was predominantly overweight, with a median body mass index (BMI) of 26.8 (24.0–29.9) kg/m^2^. Echocardiography revealed mildly reduced left ventricular systolic function and moderate left atrial enlargement, reflected by a mean left atrial volume index (LAVI) of 40.5 (29.1–47.5) mL/m^2^. Most patients exhibited low-to-moderate degrees of left atrial fibrosis. Arterial hypertension (75.9%) and hyperlipidemia (62.1%) were the most prevalent comorbidities, followed by diabetes mellitus (17.2%). The median CHA_2_DS_2_-VA score was 3, indicating a high thromboembolic risk profile. Persistent atrial fibrillation was the predominant arrhythmia subtype (56.9%), whereas paroxysmal AF was present in 41.4% of patients and long-standing persistent AF was uncommon (1.7%). A comprehensive overview of baseline patient characteristics is shown in Table 1.

### 3.2. Procedural Characteristics

Procedures were completed within a median duration of 124 min (99.2–145.0) and were associated with low fluoroscopy exposure. A median of 118 ablation points were delivered per procedure, resulting in a cumulative median ablation duration of 906 s (15.1 min). Detailed procedural characteristics, including fluoroscopy metrics, are presented in Table 2.

### 3.3. Comparison of Pacing-Based and Mapping-Based Assessment for Detection of Residual Conduction

**Figure 2:**
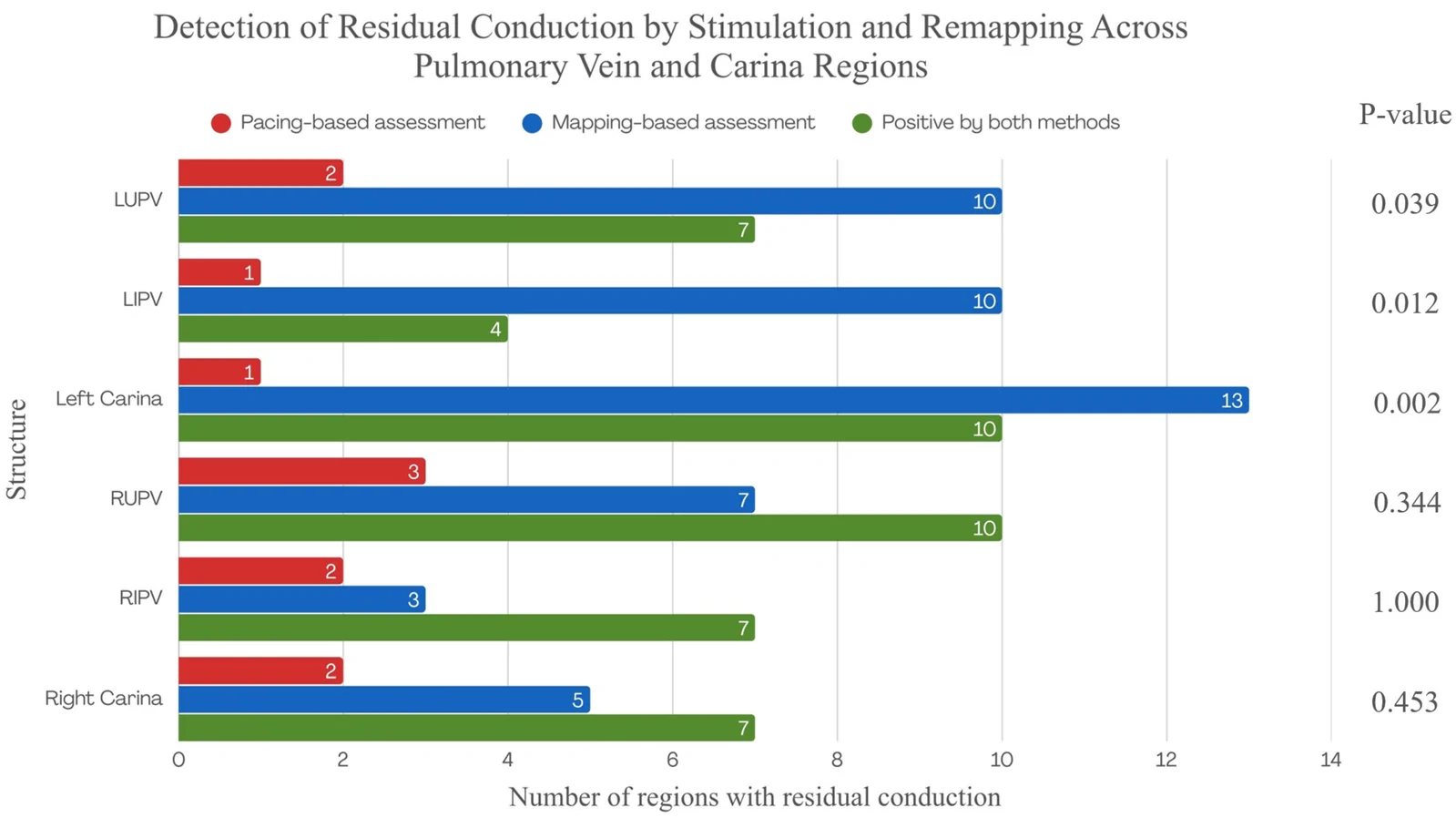
Detection of Residual Conduction by Pacing-Based and Mapping-Based Assessment Across Pulmonary Vein and Carina Regions. Numbers indicate patients with residual conduction detected by pacing-based assessment (red), mapping-based assessment (blue), or both approaches (green) at each anatomical region. *p* values compare detection by pacing-based and mapping-based assessment. LIPV, left inferior pulmonary vein; LUPV, left upper pulmonary vein; RIPV, right inferior pulmonary vein; RUPV, right upper pulmonary vein.

First-pass PVI was achieved in 26 of 58 patients (44.8%). At the individual PV level, first-pass isolation was present in 39/58 LUPVs (67.2%), 43/58 left inferior PVs (LIPVs; 74.1%), 38/58 right upper PVs (RUPVs; 65.5%), and 46/58 right inferior PVs (RIPVs; 79.3%). Residual conduction identified exclusively by high-density remapping was more frequent in the LUPV (10 vs. 2 cases detected exclusively by pacing assessment, unadjusted *p* = 0.039), the LIPV (10 vs. 1, unadjusted *p* = 0.012), and the left carina (13 vs. 1, unadjusted *p* = 0.002). After Holm correction, statistical significance was retained only for the left carina (adjusted *p* = 0.011). In contrast, no significant differences between mapping-based and pacing-based assessment were observed in the right PVs or the right carina (Figure 2). Systematic high-density remapping identified at least one additional site classified as residual conduction in 24 of 58 patients (41.4%). Additional RF applications were subsequently performed at these sites according to the procedural protocol. No minor or major procedure-related complications occurred.

Overall agreement between both assessment strategies ranged from 75.9% at the left carina to 91.4% at the RIPV, with Cohen’s κ values ranging from 0.34 to 0.69 across the individual anatomical regions. Given the limited number of discordant observations in several regions, the regional κ estimates and their 95% confidence intervals were interpreted descriptively. Detailed concordant and discordant findings and the corresponding paired statistical analyses are summarized in Table 3.

## 4. Discussion

The present study assessed the concordance between pacing-guided assessment and conventional EAM for the verification of acute PVI. Although durable PVI remains the cornerstone of catheter ablation for AF [12,13], the optimal method for confirming acute procedural success remains a matter of debate. With the emergence of newer ablation technologies, including PFA as an alternative to contemporary RF strategies, accurate intraprocedural verification of acute PVI remains an important procedural endpoint across different energy sources [14]. Conventional assessment is primarily based on the demonstration of entrance and exit block using EAM [12,15]; however, these endpoints do not invariably predict long-term lesion durability and freedom from PV reconnection [16,17]. Indeed, previous studies have demonstrated that dormant conduction and subsequent PV reconnection may persist despite apparently successful isolation, indicating that conventional remapping alone may underestimate residual conduction across ablation lesions [18,19]. In this context, adenosine administration has been investigated as an adjunctive tool to unmask dormant conduction and identify lesions at risk of later reconnection [16,18,19]. Similarly, pacing-based approaches, including pace-capture–guided assessment and other functional testing strategies, have been proposed to evaluate lesion integrity and detect sites of incomplete ablation that may not be apparent on conventional mapping alone [20,21].

Despite the growing interest in these functional assessment techniques, data directly comparing pacing-guided assessment with conventional remapping for the evaluation of acute PVI remain scarce. Furthermore, the extent to which pacing-based assessment provides incremental information beyond established electroanatomical endpoints has not been fully elucidated [10,13,20]. Therefore, the present study was undertaken to investigate the relationship between pacing-guided testing and conventional EAM in the assessment of acute PVI and to determine their potential complementarity in confirming effective and durable conduction block. Because neither strategy served as an independent reference standard, the observed differences reflect discordance in paired classifications and should not be interpreted as evidence of superior diagnostic accuracy of either assessment strategy.

The study cohort represents an elderly and clinically complex AF population characterized by a substantial burden of cardiovascular comorbidities and a predominance of persistent AF. The advanced age of the patients, along with the high prevalence of arterial hypertension, coronary artery disease, and elevated CHA_2_DS_2_-VA scores, indicates a considerable burden of cardiovascular risk factors. Compared with many contemporary PVI cohorts, in which the mean patient age typically ranges between 60 and 68 years, our population was older and exhibited a more pronounced cardiovascular risk profile [22,23,24]. This is representative of an increasingly encountered real-world AF population referred for catheter ablation.

The procedural parameters observed in the present study are comparable to those reported in contemporary RFA cohorts [25]. A median procedure duration of approximately 2 h falls within the range described for current contact force-guided PVI procedures [26]. In addition, fluoroscopy exposure remained low, with a median fluoroscopy time of approximately four minutes and a low radiation dose, reflecting the increasing use of 3D EAM systems and low-fluoroscopy workflows in contemporary electrophysiology practice [11,27]. The first-pass PVI rate of 44.8% was lower than in some previous RFA studies, such as Salló et al., who reported bilateral first-pass PVI rates of 57–80% [28]. However, direct comparison is limited by differences in acute PVI assessment. In the present study, complete isolation of all PVs and carina regions was required, and residual conduction was systematically evaluated by high-density remapping with multipolar catheters in addition to pacing testing. This more comprehensive assessment may have increased the detection of small residual conduction gaps, consistent with Vandenberk et al., who identified additional gaps by high-density remapping despite previously confirmed entrance and exit block [29].

A notable observation of the present study was the regional variability in the detection of residual conduction. Additional sites classified as residual conduction by mapping-based assessment were predominantly observed at the LUPV, LIPV, and left carina. Paired analysis further supported these regional differences, with the most robust statistical association observed at the left carina. However, the observed left-sided predominance should be considered exploratory, particularly as only the left carina remained significant after adjustment for multiple comparisons. These additional sites represent acute procedural observations and do not establish a long-term clinical benefit of remapping. Previous studies have reported heterogeneous gap distributions, including left-sided, right-sided, and more balanced patterns [29,30,31]. This variability suggests that gap localization may depend on anatomy, ablation strategy, and the method used to assess acute PVI. These additional acute procedural findings were predominantly observed in anatomically complex regions known to harbor persistent conduction despite apparently successful PVI. Previous anatomical and electrophysiological studies have highlighted the left PV ridge and carina as regions characterized by complex fiber architecture, epicardial interconnections, and variable wall thickness, factors that may complicate the creation of durable lesions and facilitate residual conduction [32,33,34]. Importantly, these additional acute procedural observations should not be regarded as clinically meaningful residual lesions, as their impact on long-term PVI durability and clinical outcomes remains uncertain. These findings should be interpreted in the context of Moser et al., who reported favorable long-term outcomes when loss of pace capture along the ablation line was used as an additional procedural endpoint. Importantly, their approach involved pacing directly along the ablation line with additional RF applications until loss of local capture was achieved, whereas the present study evaluated pacing-based and mapping-based assessment after first-pass WACA. These methodological differences may account for the apparently divergent findings [35]. In contrast, no significant differences between pacing-guided assessment and 3D EAM were observed in the right-sided PVs or at the right carina. This finding suggests that both modalities provide largely comparable information for the detection of residual conduction in these regions. One potential explanation relates to the anatomical characteristics of the left atrium. In the left-sided PVs, far-field signals originating from adjacent structures, particularly the left atrial appendage and the mitral valve region, may complicate the interpretation of local electrograms and contribute to challenges in distinguishing true residual PV conduction from surrounding atrial activity [34,36]. Although far-field signals may also be encountered within the right-sided PVs, particularly from the superior vena cava and adjacent right atrial structures, the anatomical relationship between the left atrial appendage and the left PVs is often considered especially challenging for electrogram interpretation. Consequently, differentiation between local PV potentials and far-field activity may be more demanding in the left-sided PVs than in the right-sided regions. Furthermore, the shorter distance from the transseptal puncture site to the right PVs may provide improved catheter stability and tissue contact during mapping and ablation, which could contribute to more consistent lesion formation and facilitate the identification of residual conduction [37,38]. These anatomical and procedural factors may partially explain why no significant differences in classification between the two assessment strategies were observed in the right-sided PV regions and the right carina.

### Limitations

Several limitations should be considered when interpreting the present findings. The relatively small sample size and the single-center, observational design may limit the generalizability of the results. Furthermore, the absence of randomization precludes definitive conclusions regarding the comparative performance of pacing-guided assessment and 3D EAM.

The pacing-based and mapping-based assessments were performed sequentially and were therefore not fully independent. Systematic remapping followed the initial pacing-based assessment, and suspicious mapping findings were subsequently evaluated by targeted pacing. Accordingly, the final classification of some sites may have been influenced by information obtained from the other modality. The present study should therefore be interpreted as an observational comparison of discordance between two sequential procedural assessment strategies rather than an independent diagnostic-accuracy comparison.

As the analysis was restricted to the acute verification of PVI, the impact of the observed findings on lesion durability, PV reconnection, and long-term clinical outcomes remains uncertain. Detailed segmental gap localization, post-WACA remapping duration, and the exact number of additional RF applications were not systematically captured. Accordingly, the observed regional differences should be considered hypothesis-generating and require confirmation in larger cohorts.

## 5. Conclusions

In conclusion, both pacing-guided assessment and 3D EAM are valuable tools for the acute verification of PVI. While pacing-guided testing provided comparable information in most right-sided PV regions, 3D remapping yielded additional diagnostic information in the left-sided PVs and the left carina. These findings support a complementary role of both techniques and warrant confirmation in larger studies with long-term follow-up.

## Figures and Tables

**Table 1:** Baseline Characteristics of the Study Population.

Baseline Characteristics	Total Patients (*n* = 58)
Age (years), Median [IQR]	73 [62.7–78.2]
Female, n (%)	19 (32.8)
BMI, (kg/m^2^), Median [IQR]	26.8 [24.0–29.9]
TTE LVEF (%), Median [IQR]	48.0 [41.5–59.0]
TTE LA Diameter (cm), Median [IQR]	4.8 [4.3–5.5]
LAVI (mL/m^2^), Median [IQR]	40.5 [29.1–47.5]
LA Pressure (mmHg), Median [IQR]	10.0 [9.0–13.0]
Degree of LA Fibrosis, Median [IQR]	1 [0–2]
0, n (%)	21 (36.2)
I, n (%)	18 (31.0)
II, n (%)	16 (27.6)
III, n (%)	3 (5.2)
Arterial Hypertension, n (%)	44 (75.9)
Hyperlipidemia, n (%)	36 (62.1)
Coronary Artery Disease, n (%)	33 (56.9)
Diabetes Mellitus, n (%)	10 (17.2)
Chronic Obstructive Pulmonary Disease, n (%)	2 (3.4)
Obstructive Sleep Apnea Syndrome, n (%)	6 (10.3)
Positive Family History, n (%)	14 (24.1)
Smoking, n (%)	12 (20.7)
CHADS-VA Score, Median [IQR]	3 [2.0–4.0]
Type of Atrial Fibrillation	
Paroxysmal, n (%)	24 (41.4)
Persistent, n (%)	33 (56.9)
Long-standing persistent, n (%)	1 (1.7)

BMI, body mass index; IQR, Interquartile Range; LA, left atrium; LAVI, left atrial volume index; LVEF, left ventricular ejection fraction; TTE, transthoracic echocardiography.

**Table 2:** Procedural Characteristics of the Study Population.

Procedural Characteristics	Total Patients (*n* = 58)
Procedure Duration (min), Median [IQR]	124 [99.2–145.0]
Fluoroscopy Time (s), Median [IQR]	214 [0–441.6]
Fluoroscopy Dose (cGy·cm^2^), Median [IQR]	212.3 [0–666.6]
Total Number of Ablation Points, Median [IQR]	118 [56.5–146.5]
Total Ablation Duration (s), Median [IQR]	906 [604.5–1188.5]

IQR, interquartile range; min, minutes; s, seconds.

**Table 3:** Regional agreement between pacing-based and mapping-based assessment of residual conduction after first-pass WACA.

Region	Pacing	Mapping	Both Residual	Both Isolated	Agreement, %	Cohen’s κ (95% CI)	Exact McNemar *p*	Holm- Adjusted *p*
LUPV	2	10	7	39	79.3	0.42 (0.16–0.68)	0.039	0.154
LIPV	1	10	4	43	81.0	0.34 (0.06–0.62)	0.012	0.059
Left carina	1	13	10	34	75.9	0.45 (0.23–0.67)	0.002	0.011
RUPV	3	7	10	38	82.8	0.55 (0.31–0.79)	0.344	1.000
RIPV	2	3	7	46	91.4	0.69 (0.43–0.94)	1.000	1.000
Right carina	2	5	7	44	87.9	0.59 (0.33–0.86)	0.453	1.000

Results are shown separately for each pulmonary vein and ipsilateral carina, including concordant and discordant classifications, overall agreement, Cohen’s κ, and exact McNemar testing for paired observations. “Both residual” indicates concordant classification of residual conduction by both assessment strategies, whereas “Both isolated” indicates concordant classification of complete isolation by both strategies. CI, confidence interval; LIPV, left inferior pulmonary vein; LUPV, left upper pulmonary vein; RIPV, right inferior pulmonary vein; RUPV, right upper pulmonary vein; WACA, wide antral circumferential ablation.

## Data Availability

The data presented in this study are available on request from the authors. The data are not publicly available due to data privacy laws.

## Abbreviations

The following abbreviations are used in this manuscript:

3D	Three-dimensional
ACT	Activated clotting time
AF	Atrial fibrillation
BMI	Body mass index
EAM	Electroanatomical mapping
IQR	Interquartile ranges
LA	Left atrium
LAVI	Left atrial volume index
LIPV	Left inferior pulmonary vein
LUPV	Left upper pulmonary vein
LVEF	Left ventricular ejection fraction
Min	Minutes
MV	Mitral valve
PV	Pulmonary vein
PVI	Pulmonary vein isolation
RFA	Radiofrequency ablation
RIPV	Right inferior pulmonary vein
RUPV	Right upper pulmonary vein
S	Seconds
SD	Standard deviation
TTE	Transthoracic echocardiography
UCH	Unfractionated heparin
WACA	Wide antral circumferential ablation
